# Effect of Medium pH on Antibiotic Activity against Syrian *Brucella* spp. Isolates

**Published:** 2013-09

**Authors:** Ayman Al-Mariri, Mazen Safi

**Affiliations:** Department of Molecular Biology and Biotechnology, Atomic Energy Commission, Damascus, Syria

**Keywords:** Antibacterial, Antibiotics, *Brucella*, Zoonotic, Quinolone

## Abstract

**Background:** Brucellosis is an endemic zoonosis in Syria, affecting large numbers of animals. There are an increasing number of cases in humans. *Brucella* is a facultative intracellular pathogen, a small, non-motile, Gram-negative coccobacillus, which causes abortion in domestic animals and a febrile illness in humans.

**Methods: **One hundred isolates collected from different Syrian regions were confirmed to be *Brucella melitensis* by biochemical tests. The minimum inhibitory concentration (MIC) of 6 antibiotics, alone and in combination, was determined at pH 7.0 and pH 5.0.

**Results: **Ciprofloxacin and sparfloxacin were the most effective antibiotics tested at either pH value. In contrast, rifampicin had low activity and streptomycin was ineffective at either pH value. A combination of rifampicin-doxycycline revealed the highest synergistic activity at both test pH values (against 19/24 and 17/24 isolates, respectively) in vitro. Antagonistic activities were observed using a ciprofloxacin-streptomycin combination (against 9/24 and 13/24 isolates, respectively) as well as a ciprofloxacin-tetracycline combination (against 6/24 and 9/24 isolates, respectively). No differences were observed at both test pH values, when combining a Quinolone with rifampicin or doxycycline.

**Conclusion: **Combination of a Quinolone with doxycycline demonstrated good *in vitro* activity against *B. melitensis*. Further in vivo studies are necessary to support this suggestion.

## Introduction

Brucellosis is a zoonotic disease with worldwide distribution, but it is most frequent in the Mediterranean basin and South America.^1^ Because the bacteria are intracellular, successful treatment requires antibiotics with good cellular penetration. Different regimens have been universally applied in clinical practice.^2^ The most recent recommendation by the World Health Organization (WHO) for the treatment of acute brucellosis in adults suggested a dose of 600 to 900 mg rifampicin and 200 mg doxycycline daily for a minimum of 6 weeks.^3^ Empirically, it has been suggested that a previous regimen of streptomycin in combination with oral tetracycline results in fewer relapses than a rifampicin-doxycycline combination.^4^^,^^5^ In addition, rifampicin monotherapy is the main recommended option for treatment during pregnancy, and a combination of rifampicin with Trimethoprim-Sulfamethoxazole is the suggested regimen for children.^6^^,^^7^ Triple-antibiotic combinations have been found to be of value in some cases of *brucella* endocarditis, meningitis, and spondylitis.^8^^-^^10^ Quinolone treatment has been shown to be a therapeutic alternative, and various combinations that incorporate ciprofloxacin and ofloxacin have been tried clinically, yielding similar efficacy to that of the classic regimens.^11^ Only in vitro observations exist for Moxifloxacin and Levofloxacin.^12^ Fluoroquinolones and newer Macrolides have good anti-brucellosis activity in vitro^13^^-^^15^ and reach high intracellular concentrations, but their *in vitro* activity may predict efficacy poorly because *Brucella* survive in compartments that are inaccessible or hostile to antimicrobial activity. These include the phagolysosomes of macrophages, where the pH may be as low as 5. In consequence, specialized agents that are able to penetrate the macrophages and function within their cytoplasm are required for the treatment of brucellosis.^16^ Acidity impairs the activity of Quinolones and Macrolides.

The aim of this study was to evaluate, in vitro, the effect of medium acidity on the activities of some antibiotics, alone and in combination, against some Syrian *Brucella* *melitensis* isolates collected from different provinces. The single antibiotics were doxycycline, rifampicin, tetracycline, streptomycin, ciprofloxacin, and sparfloxacin, whereas the antibiotic combinations were rifampicin-tetracycline, rifampicin-doxycycline, rifampicin-ciprofloxacin, rifampicin-sparfloxacin, rifampicin-streptomycin, ciprofloxacin-tetracycline, ciprofloxacin-doxycycline, ciprofloxacin-streptomycin, and ciprofloxacin-sparfloxacin. 

## Materials and Methods

*Microorganisms and Growth Conditions*

One hundred *B. melitensis* isolates were collected prospectively between 2004 and 2007 from bovine and ovine milk from different Syrian provinces. These provinces were divided into four regions, as follows: Northern (including Al-Hasakah, Deer-Alzour, Al-Rakah, and Aleppo Provinces); Central (including Edleb, Hamaa, and Homs Provinces); Coastal (including Tartous and Lattakia Provinces); and Southern (including Al-Quonaitra, Daraa, Al-Souaida, Damascus, and Damascus rural Provinces). Bacteria were isolated from the milk cultures at the Immunology/Microbiology Laboratory, Atomic Energy Commission of Syria (AECS).^17^ They were identified to the species level via conventional methods (the requirement for CO_2_ for growth, production of H_2_S, urease production, sensitivity to thionine and basic fuchsin, and agglutination with specific antiserum). A class II biological safety cabinet was used. During the work, the laboratory workers were wearing impermeable protective clothes, gloves, and a face mask. 

*Minimum Inhibitory Concentration Determination at Different pH Values*

In order to estimate the antibiotics susceptibility, the well broth microdilution method was utilized with 96-well plates (TPP, Switzerland). The antibiotics (i.e. doxycycline [Sigma, St. Louis, MO, USA], rifampicin [Sigma], tetracycline [Sigma], streptomycin [Sigma], ciprofloxacin [Bayer, Istanbul, Turkey], and sparfloxacin [Sigma] were diluted twofold in *Brucella* broth^®^ (Acumedia, Michigan, USA) and adjusted to pH 7.0 and pH 5.0. The wells were inoculated with 10^6^ CFU of the bacteria (in a 0.2-ml final volume). The incubation period was 48 h at 37^°^C. The lowest concentration that completely inhibited visual growth was recorded and interpreted as the minimum inhibitory concentration (MIC). MIC testing was performed according to the recommendations of the Clinical Laboratory Standards (CLSI).^18^ The range of the concentrations assayed for each antibiotic was 0.125 to 128 μg/ml. *Escherichia coli* ATCC 25922 and *Staphylococcus aureus* ATCC 25923 served as controls. 

*Antibiotic Combination Studies*

Twenty-four of the 100 *Brucella* isolates (six isolates from each region) were randomly chosen to evaluate the antibiotic combination effects. Checkerboard titrations were used at pH 5.0 and pH 7.0 in the same conditions to assess the MICs and to evaluate the activities of the 9 above-mentioned antibiotic combinations. Strains showing synergy, a marked additive effect, or antagonism were retested using the broth dilution method, with each well containing the final antibiotic concentration used in the plates. In this checkerboard test, the sum of the fractional inhibitory concentration (∑ FIC) was calculated as described previously.^19^^,^^20^ The ∑ FIC was classified as follows: synergistic≤0.75; additive from 0.75 to 1; indifferent from 1 to 2; and antagonistic≥2.

*Statistical Methods*

All the analyses were conducted with version 4.0 of GraphPad Prism. Fisher’s exact test was used to make a comparison between the susceptible and non-susceptible isolates toward each antibiotic at pH 5.0 and pH 7.0. A P value≤0.05 was considered statistically significant. 

## Results

Table 1 demonstrates that, under the conditions of our study, ciprofloxacin and sparfloxacin were the most effective individual antibiotics against *B. melitensis *from any Syrian region (Northern, Central, Coastal, and Southern), with the MICs ranging from 0.125 μg/ml to 8 μg/ml. Doxycycline and tetracycline were less effective than ciprofloxacin or sparfloxacin, with the MICs ranging from 0.5 μg/ml to 16 μg/ml for the former and from 0.25 μg/ml to 16 μg/ml for the latter; however, they were less effective against the *Brucella* isolates from the Coastal region. Rifampicin had the lowest activity against *Brucella *from the Northern and the Coastal regions; the MICs ranged from 32 μg/ml to 64 μg/ml at both pH values. Table 1 also reveals that the overall susceptibility rates of ciprofloxacin, doxycycline, and sparfloxacin against all the isolates were 97%, 92%, and 98% at pH 5.0; and 98%, 94%, and 99% at pH 7.0, respectively. Fifty-one isolates were resistant to rifampicin at both pH conditions (particularly the isolates from the Northern (n=28) and Coastal (n=18) regions), whereas 39 and 27 isolates were resistant to tetracycline at pH 5.0 and pH 7.0, respectively. No significant differences were observed regarding each individual antibiotic between pH 5.0 and pH 7.0, with the exception of the effect of tetracycline against the Southern region isolates, where the susceptibility was decreased at pH 5.0 compared with that at pH 7.0 (17 vs. 27 isolates; P<0.0007). Finally, 100% of the isolates were resistant to streptomycin. 

**Table 1:** Effect of medium pH levels on MIC_range_ and MIC_90_ and the susceptibility percentage (Susc.%) of some antibiotics against *B. melitensis* isolates collected from different Syrian regions

**Streptomycin**		**Sparfloxacin**		**Rifampicin**		**Tetracycline**		**Doxycycline**		**Ciprofloxacin**		**Antibiotics**	
**pH7**	**pH5**	**pH7**	**pH5**	**pH7**	**pH5**	**pH7**	**pH5**	**pH7**	**pH5**	**pH7**	**pH5**		**Regions (number of isolates)**
64->128	>128	0.125-0.5	0.25-2	32-64	32-64	2-16	2-16	0.5-8	0.5-8	0.125-0.5	0.125-1	MIC_range_ (µg/ml)	Northern region (30)
>128	>128	0.5	2	64	64	16	16	4	8	0.5	1	MIC_90_ (µg/ml)	
0% (0)	0% (0)	100% (30)	100% (30)	7% (2)	7% (2)	54% (16)	50% (15)	93% (28)	90% (27)	100% (30)	100% (30)	Susc. % (N)	
>128	>128	0.5-2	0.5-2	2-4	2-4	0.25-1	0.25-8	0.5-1	0.5-2	0.5-1	0.5-1	MIC_range_ (µg/ml)	Central region (20)
>128	>128	2	2	4	4	1	8	1	1	1	1	MIC_90_ (µg/ml)	
0% (0)	0% (0)	100% (20)	100% (20)	90% (18)	90% (18)	95% (19)	90% (18)	100% (20)	100% (20)	100% (20)	100% (20)	Susc. % (N)	
>128	>128	1-4	1-4	32-64	32-64	8-16	8-16	8-16	4-8	1-8	1-8	MIC_range_ (µg/ml)	Coastal region (20)
>128	>128	4	4	64	64	16	16	16	8	4	8	MIC_90_ (µg/ml)	
0% (0)	0% (0)	95% (19)	95% (19)	10% (2)	10% (2)	55% (11)	55% (11)	85% (17)	90% (18)	90% (18)	90% (18)	Susc. % (N)	
64->128	>128	0.25-4	0.25-4	2-8	4-8	1-8	1-16	1-4	0.5-8	0.25-2	2-4	MIC_range_ (µg/ml)	Southern region (30)
>128	>128	2	4	8	8	8	16	4	8	2	4	MIC_90_ (µg/ml)	
0% (0)	0% (0)	100% (30)	97% (29)	90% (27)	90% (27)	90% (27)	57% (17)	97% (29)	90% (27)	100% (30)	97% (29)	Susc. % (N)	
0% (0)	0% (0)	99%	98%	49%	49%	73%	61%	94%	92%	98%	97%	Susc. %	All regions

MIC: Minimum inhibitory concentration; Susc.%: Percentage of susceptible isolates in each region; (N): Number of susceptible isolates in each region

**Figure 1:**
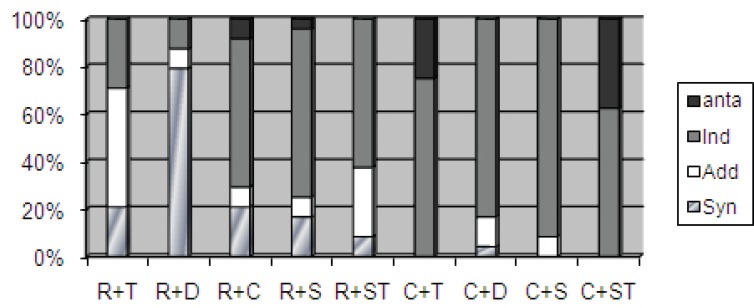
This is a representation of the activity of the antibiotic combinations at pH 7.0. R: Rifampicin; T: Tetracycline; D: Doxycycline; C: Ciprofloxacin; S: Sparfloxacin; ST: Streptomycin; Anta: Antagonism; Ind: Indifference; Add: Additive; Syn: Synergy

**Figure 2:**
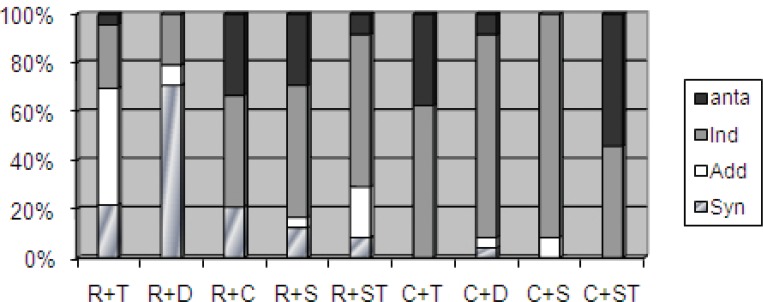
This figures illustrates the activity of the antibiotic combinations at pH 5.0. R: Rifampicin; T: Tetracycline; D: Doxycycline; C: Ciprofloxacin; S: Sparfloxacin; ST: Streptomycin; Anta: Antagonism; Ind: Indifference; Add: Additive; Syn: Synergy

Figures 1 and 2 present the data on the effects of the antibiotic combinations at pH 7.0 and pH 5.0, respectively, on 24 selected *Brucella* isolates. The rifampicin-doxycycline combination showed a synergistic activity against 19 and 17 isolates at pH 7.0 and pH 5.0, respectively. The ciprofloxacin-doxycycline, ciprofloxacin-sparfloxacin, and rifampicin-sparfloxacin combinations were indifferent against 20, 22, and 17 isolates at pH 7.0, respectively; and against 20, 22, and 13 isolates at pH 5.0, respectively. The rifampicin-tetracycline and rifampicin-streptomycin combinations showed additive activities against 12 and 7 isolates at pH 7.0; and against 12 and 5 isolates at pH 5.0, respectively. However, the ciprofloxacin-streptomycin and ciprofloxacin-tetracycline combinations demonstrated antagonistic activity against 9 and 6 *Brucella* isolates at pH 7.0; and against 13 and 9 isolates at pH 5.0, respectively. 

## Discussion

*Brucella *spp. infect macrophages replicating within the phagolysosomes at a pH of 5.0.^16^ Theoretically, antibiotics that are able to penetrate the phagolysosomal compartment and function under acidic conditions could be used as monotherapy for the treatment of *Brucella*. However, in practice, neither doxycycline nor rifampicin (both of which meet these criteria) is effective as a monotherapeutic agent.^1^^,^^14^ Garcia-Rodriguez et al*.*^21^ found a two to fourfold decrease in the activity of Quinolones against *Brucella* at a pH of 5.0 compared to a pH of 7.0. In their study, all the Quinolones exhibited very low bactericidal activities against the 21 tested strains, regardless of the pH, with a MBC_90s_ (minimal bactericidal concentration) of ≥8 mg/liter. In addition, Akova et al.^20^ revealed that only rifampicin and doxycycline retained sufficient activity against *Brucella *at a pH of 5.0, in contrast to the other tested antibiotics. The authors showed that the rifampicin activity increased two to eightfold at the acidic pH. 

Antibiotic combination studies have revealed an absence of synergism between Quinolones and other antibiotics against *B. melitensis*.^22^^,^^23^ Akova et al.^20^ studied the combination of ofloxacin-rifampicin against 20 isolates at pH 7.0 and 5.0 and found antagonism in 17 isolates and indifference in 3 isolates at pH 7.0. In contrast, at pH 5.0, this combination exhibited antagonism, indifference, additive effects, and synergy in 7, 8, 1, and 4 isolates, respectively. The combination of rifampicin-doxycycline was found to be the most synergistic.

On the hand, and in their efforts to evaluate the susceptibility of *B. melitensis* against several antibiotics, Qadri et al.^24^ reported cross-resistance of *B. melitensis* isolates to all Quinolones noted after therapy with ciprofloxacin. A good activity of ciprofloxacin has been reported in many *in vitro* studies.^25^^,^^26^ In addition, Baykam et al*.*^27^ in a study performed in Turkey and Dimitrov et al.^28^ in a study performed in AL Kuwait^28^ found that all their isolates were susceptible to ciprofloxacin, but 9.6% and 8% of the isolates were resistant to rifampicin in vitro, respectively.

In our study, we detected no differences regarding the individual antibiotic activity when we tested ciprofloxacin (MIC_range_: 0.125-8 μg/ml at both pH levels) or sparfloxacin (MIC_range_: 0.125-4 μg/ml at pH 7.0, and 0.25-4 μg/ml at pH 5.0) against the *Brucella* isolates from any Syrian region at either pH value. At pH 5.0, the tetracycline activity was reduced in the Central region isolates and its susceptibility in the Southern region was decreased at pH 5.0 compared with that at pH 7.0 (P<0.0007). The rifampicin activity was very low in the Coastal and the Northern regions at both pH levels (MIC_range_: 32-64 μg/ml). In addition, rifampicin-resistant isolates were observed in these two regions (18 and 28 resistant isolates, respectively). However, one of the most unexpected results in this study was the very poor activity of streptomycin against all the *Brucella* isolates (MIC_range_>128 μg/ml), which has not been published previously.^20^^,^^22^ We suggest that this resistance to streptomycin could have been developed as a result of the aggressive administration of this antibiotic in the treatment for all causes of bovine udder infection cases in Syria. 

Moreover, in another study performed in our laboratory, we found that the MIC_range_ was 0.125-16 μg/ml for ofloxacin and 0.125-8 μg/ml for Levofloxacin, indicating the good activity of these two antibiotics against Syrian *Brucella* isolates (data not shown).

No antagonism was seen with the rifampicin-doxycycline or rifampicin-tetracycline combinations at both pH conditions, while antagonism was clear when the ciprofloxacin-tetracycline and ciprofloxacin-streptomycin combinations were assessed. In addition, antagonism increased at pH 5.0 compared to pH 7.0 when rifampicin-ciprofloxacin and particularly rifampicin-sparfloxacin combinations were used. No synergic or additive effects were observed when we applied the new combinations at both pH conditions, whereas the rifampicin-doxycycline combination was the most synergistic at both pH degrees.

Nevertheless, the return of brucellosis during the use of Quinolone has been mentioned previously. A prospective study by al Sibai et al.^29^ reported high probabilities of brucellosis relapse after monotherapy with ciprofloxacin (26.7%). On the other hand, in a retrospective study by Tekkok et al.^30^ ofloxacin monotherapy led to a higher probability of brucellosis relapse than the ofloxacin-rifampicin combination in a small number of patients with spondylitis.^30^ Aygen et al.^31^ revealed that in 480 patients with various forms of brucellosis, the probabilities of relapse for the various treatment regimens were 4.6% for the patients who received non-Quinolone regimens and 17.9% for those who received Quinolone-based regimens (21.4% for ciprofloxacin monotherapy and 14.3% for the combinations of Quinolones with other antibiotics).

## Conclusion

Our results suggest the presence of a good activity of ciprofloxacin and sparfloxacin, with the exception of the rifampicin-sparfloxacin combination at pH 5 alone and with combination with other traditional antibiotics used in the treatment of brucellosis infection, in vitro, against Syrian *Brucella* isolates collected from different provinces. The activity of rifampicin in this study was mediocre, even though it is considered a front-line treatment used in brucellosis therapy. However, a combination of doxycycline and rifampicin enhanced the activity of rifampicin in both pH values. Unfortunately, streptomycin did not have any activity against these isolates.

Finally, if the treatment with Quinolones is opted for, care should be taken because the consumption of Quinolone alone can probably cause the relapse of *Brucella* disease. Then, when it is used instead of rifampicin, doxycycline should be applied simultaneously.

Further and more specific studies, in vivo, are recommended to determine the efficacy of these Quinolones in the treatment of brucellosis infections. If rifampicin could be replaced by ciprofloxacin and sparfloxacin, then rifampicin use could be restricted solely to the treatment of tuberculosis, which is regarded as a big challenge in Syria.
